# Supplementary material for: Comprehensive Annotation and Functional Exploration of MicroRNAs in Lettuce
Source: Front Plant Sci. 2021 Dec 24;12:781836. doi: 10.3389/fpls.2021.781836 (PMC8739914; doi:10.3389/fpls.2021.781836)
Supplement: Supplementary file 2 [file Data_Sheet_8.ZIP › GSM2230747.plot/Lsa-miRN48_Lsat_1_v5_gn_9_6621.1_3256_TPlot.pdf]

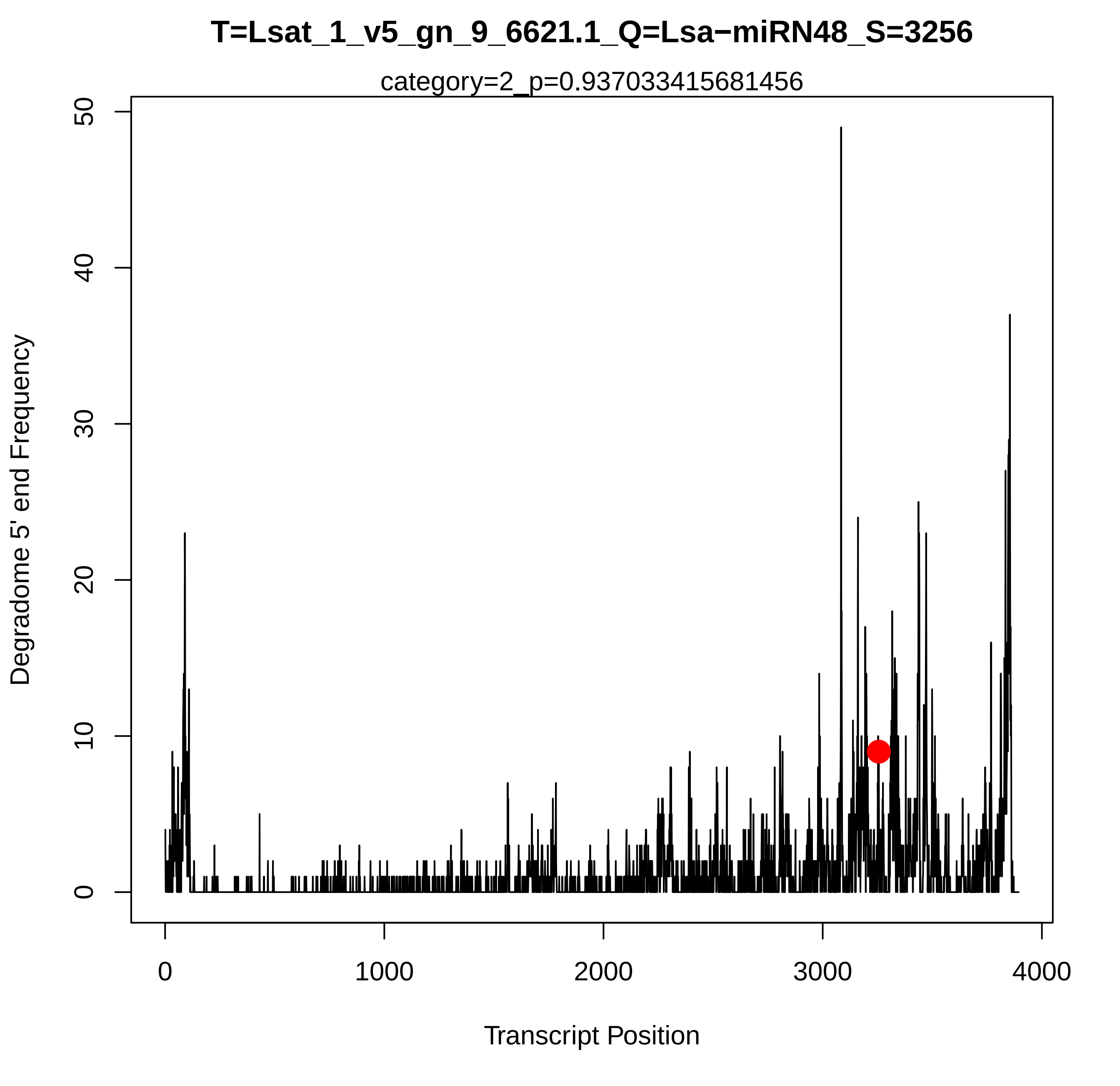

T=Lsat_1_v5_gn_9_6621.1_Q=Lsa−miRN48_S=3256
category=2_p=0.937033415681456
Degradome 5' end Frequency
Transcript Position
0
10
20
30
40
50
0
1000
2000
3000
4000
